# Burnout syndrome among dental students: a short version of the "Burnout Clinical Subtype Questionnaire" adapted for students (BCSQ-12-SS)

**DOI:** 10.1186/1472-6920-11-103

**Published:** 2011-12-12

**Authors:** Jesus Montero-Marin, Francesca Monticelli, Marina Casas, Amparo Roman, Inmaculada Tomas, Margarita Gili, Javier Garcia-Campayo

**Affiliations:** 1 Department of Psychiatry. Faculty of Medicine. Zaragoza. Spain; 2 Department of Surgery, Gynecology and Obstetrics. Faculty of Health and Sports. Huesca. Spain; 3 Department of Stomatology. Faculty of Medicine and Dentistry. Santiago de Compostela. Spain; 4 Department of Psychology. Faculty of Psychology. Mallorca. Spain; 5 REDIAPP "Research Network on Preventative Activities and Health Promotion" (RD06/0018/0017

**Keywords:** burnout, subtypes, BCSQ-12-SS, dental students, factorial validity

## Abstract

**Background:**

Burnout has been traditionally defined in relation to the dimensions of "exhaustion", "cynicism", and "inefficiency". More recently, the Burnout Clinical Subtype Questionnaire (BCSQ-12) further established three different subtypes of burnout: the "frenetic" subtype (related to "overload"), the "under-challenged" subtype (related to "lack of development"), and the "worn-out" subtype (related to "neglect"). However, to date, these definitions have not been applied to students. The aims of this research were (1) to adapt a Spanish version of the BCSQ-12 for use with students, (2) to test its factorial validity, internal consistency, convergent and discriminant validity, and (3) to assess potential socio-demographic and occupational risk factors associated with the development of the subtypes.

**Method:**

We used a cross-sectional design on a sample of dental students (n = 314) from Santiago and Huesca universities (Spain). Participants completed the Burnout Clinical Subtype Questionnaire Student Survey (BCSQ-12-SS), the Maslach Burnout Inventory Student Survey (MBI-SS), and a series of socio-demographic and occupational questions formulated for the specific purpose of this study. Data were subjected to exploratory factor analysis (EFA) using the principal component method with varimax orthogonal rotation. To assess the relations with the criterion, we calculated the Pearson correlation coefficient (r), multiple correlation coefficient (R_y.123_), and the coefficient of determination (R^2^_y.123_). To assess the association between the subtypes and the socio-demographic variables, we examined the adjusted odds ratio (OR) obtained from multivariate logistic regression models.

**Results:**

Factorial analyses supported the theoretical proposition of the BCSQ-12-SS, with α-values exceeding 0.80 for all dimensions. The "overload-exhaustion" relation was r = 0.59 (p < 0.001), "lack of development"-"cynicism", r = 0.49 (p < 0.001), "neglect"-"inefficiency", r = 0.47 (p < 0.001). The "overload"-"lack of development" relation was r = 0.21 (p < 0.001), "overload"-"neglect", r = 0.20 (p < 0.001), and "lack of development"-"neglect", r = 0.38 (p < 0.001). The BCSQ-12-SS explained 38.44% of the variability in "exhaustion", (R_y.123 _= 0.62), 30.25% in "cynicism" (R_y.123 _= 0.55), and 26.01% in "inefficiency" (R_y.123 _= 0.51). "Hours spent on studying" was found to be associated with "overload" (p = 0.001), "campus" with "lack of development" (p = 0.013), and ""failed subjects" with "neglect" (p = 0.011).

**Conclusions:**

The results support the definition of burnout as established by the BCSQ-12-SS. As such, the BCSQ-12-SS can be used for the recognition of clinical profiles and for the suggestion of potential intervention strategies specific to the characteristics of each particular case.

## Background

Chronic stress in the work environment is a fundamental risk factor for developing burnout syndrome [1]. Burnout is a physical response that an individual might experience when he or she fails to regulate stress effectively, and could have serious consequences on one's health [2].

Traditionally, burnout syndrome has been defined as a situation in which the affected person experiences feelings of "emotional fatigue", "depersonalisation", and "lack of personal achievement". "Emotional fatigue" prevents workers from engaging in their work at an emotional level due to their perceived lack of energy. "Depersonalisation" refers to the development of negative feelings and behaviour towards other people, and often involves blaming others for one's own problems. "Lack of achievement" refers to the tendency to assess one's own ability negatively and involves feelings of unhappiness and dissatisfaction [3]. However, to be able to apply the definition of burnout across all kinds of occupations, this syndrome has been redefined and standardized on three dimensions: "exhaustion", "cynicism", and "inefficiency". "Exhaustion", operating at the emotional level, refers to the feeling of not being able to give any more of oneself to work. "Cynicism" is shown in distancing behaviours towards work, customers, and co-workers. Finally, "inefficiency" refers to one's feelings of inadequacy and incompetence when performing tasks at work [4].

Although burnout syndrome tends to be more prevalent in assistance or service professions, it has been observed in all types of occupations [5]. Among university students [6], burnout syndrome has been found to be especially prevalent in those training for health careers, such as medicine [7] and nursing [8]. In particular, past studies have found dentists to be highly likely to develop burnout due to the nature of their clinical work [9-11]. In addition, both the education and practice of dentists have been well-documented as sources of stress. For example, in the course of their education, 10% of dental students suffered from serious levels of "emotional fatigue", 28% showed symptoms of "depersonalisation", and 17% felt a "lack of personal achievement" [12]. Burnout syndrome has been found to be most severe when dentists make their first step into the professional world. Therefore, dental universities have been advised to incorporate the instruction of stress management skills into their programmes [13].

Recently, a newer and broader definition of burnout has been developed by our group based on research using the "Burnout Clinical Subtype Questionnaire" (BCSQ-36). This new definition, only validated among samples of workers, differentiates between three clinical subtypes of burnout that vary on the level of dedication at work. The "frenetic" subtype, characterised by investing a large amount of time in working, is typical of people who are very involved, ambitious, and overloaded. The "under-challenged" subtype, characterised by feelings of indifference, boredom, and lack of personal development, is typical of people who perform mechanical tasks. The "worn-out" subtype is characterised by the feeling of losing control over outcomes, the perceived lack of recognition of one's own efforts, and the giving up of responsibilities. The "worn-out" subtype is influenced by the rigidity of the organisational structure at work [14-17].

The dimensions 'overload', 'lack of development and 'neglect', belonging to the "frenetic", underchallenged" and "worn-out" subtypes, respectively, comprise a definition of burnout that comes close to the standard and typological approaches [18]. This brief definition, also developed by our research group, is operationalized by means of the short version of the Burnout Clinical Subtype Questionnaire or BCSQ-12. 'Overload' refers to individuals' feeling of risking health and personal life in the pursuit of good results, and is significantly associated with 'exhaustion'; 'lack of development' refers to the absence of personal growth experiences for individuals together with their desire take on other jobs where they can better develop their skills, and is significantly associated with 'cynicism'; 'neglect refers to individuals' disregard as a response to any difficulty, and is strongly associated with 'inefficacy'.

The dimensions established in the BCSQ-12 have proven useful for the quick recognition of burnout subtypes with criterion validity [19]. However, these dimensions have not been tested among students. It is worth investigating whether the model based on BCSQ-12 is valid among dental students, given the characteristics of this population as well as the possibility of enabling more specific assessments and interventions among a population highly affected by burnout. Therefore, the aims of the present study included adapting the BCSQ-12 for use with students, evaluating its factorial structure, internal consistency, convergent and discriminant validity, and contrast the socio-demographic and occupational risk factors associated with the development of each burnout subtype.

## Method

### Design and study population

A cross-sectional design was used, with analyses based on self-reported data collected from two different sites. The pool of potential participants consisted of dental students (N = 378) attending the Spanish universities of Huesca (N_H _= 136) and Santiago de Compostela (N_S _= 242) in the 2010-11 academic year. Of all the potential participants, 314 students completed and returned the surveys, achieving a response rate (RR) of 83.07%. The sample size exceeded the evaluation criterion of construction and composition validity that was necessary given the number of covariates [20,21], which lent psychometric adequacy to the analysis. We did not find significant differences in the response rate between students from the two universities (p = 0.092). Neither did we find differences between the participants and non-participants in terms of age (p = 0.493), gender (p = 0.322) or year of study (p = 0.102).

### Procedure

A clinical psychologist provided instructions to the lecturers at both universities on how to administer the questionnaires. Prior to beginning the study, participants provided their informed consent by reading and approving the objectives of the study, the participants at whom it was targeted, the voluntary nature of study participation, the potential benefits/risks of the study, and the total confidentiality of the data, as described on the first page of the protocol. The survey was administered by lecturers using time between classes during the last week of May 2011, two weeks before the exam period began. Completed questionnaires were gathered in sealed envelopes to ensure anonymity. The project was approved by the Aragón Regional Ethical Committee.

### Measurements

#### Socio-demographic and occupational factors

First, we collected data on the following socio-demographic characteristics of the participants: age, gender, whether one was in a stable relationship ("yes" vs. "no"), children ("yes" vs. "no"), campus ("Huesca" vs. "Santiago"), distance from family home in kilometres, place of residence during the year ("with parents", "dormitory", "shared flat", "private flat"), scholarship ("yes" vs. "no"), perceived parental support for one's studies ("insufficient", "good", "very good"), weekly time spent on studying, failed subjects over the previous exam period ("none", "one", "two or more"), job ("yes" vs. "no"), and year of study ("first", "second", "third", "fourth", "fifth").

#### Burnout Clinical Subtype Questionnaire Students Survey (BCSQ-12-SS)

Then participants responded to a short version of the "Burnout Clinical Subtype Questionnaire", or BCSQ-12 [18], adapted for use with students ("Burnout Clinical Subtype Questionnaire Students Survey", or BCSQ-12-SS). The adaptation procedure consisted of changing all references to work into references to student activity (Additional File 1 shows the Spanish version of the questionnaire and Additional File 2 shows the English version, although only the Spanish version was used in this study. The English version has not yet been the subject of a validation study). The BCSQ-12-SS consists of 12 items that were evenly distributed among the dimensions: "overload" (e.g., "I think I invest more than is healthy in my commitment to my studies"), "lack of development" (e.g., "I would like to study something else that would be more challenging to my abilities"), and "neglect" (e.g., "When the results of my studies are not good at all, I stop making an effort"). The subjects had to indicate the point to which they agreed with each item using a Likert-type scale with 7 response options ranging from 1 ("completely disagree") to 7 ("completely agree"). Results are presented in scalar scores. High internal consistency was achieved for each dimension of the original BCSQ-12, with adequate criterion validity [16,18].

#### Maslach Burnout Inventory Student Survey (MBI-SS)

Finally, an adaptation in Spanish of the Maslach Burnout Inventory General Survey or MBI-GS [4], version for students, the Maslach Burnout Inventory Student Survey or MBI-SS [6], was administered. This adaptation consists of 15 items where the references to work are changed for references to study. Five items corresponded to the "exhaustion" dimension (e.g.: "I am emotionally exhausted by this career"), 4 items corresponded to the "cynicism" dimension (e.g.: "I lost enthusiasm for my career"), and 6 items corresponded to the "efficiency" dimension (e.g.: "In my opinion, I am a good student"). Participants responded on a Likert-type scale with 7 response options that ranged from 0 ("never") to 6 ("always"). Results are presented in scalar scores. Both the factorial solution of the scale, and the reliability of the dimensional components, have proven to be consistent, with α-values ≥0.74 [6].

### Data analysis

The continuous socio-demographic and occupational variables were recoded into 3 levels, which were introduced in the analysis as dummy variables as follows: age (" < 20 years old", "20 to 22 years old", " > 22 years old"), distance from family home (' < 75 Km', '75-150 Km', ' > 150 Km') and weekly time studying (' < 30 hours, '30-40 hours, ' > 40 hours). We conducted a descriptive analysis of participant characteristics in the entire sample and separately for each campus ("Huesca" vs. "Santiago") using frequencies, percentages and χ^2 ^statistics to assess potential differences. Means, standard deviation, minimum and maximum statistics were calculated for each item in the BCSQ-12-SS

We tested the factor structure of the BCSQ-12-SS using an exploratory factorial analysis (EFA), trough principal component method with varimax orthogonal rotation. We performed a series of preliminary analyses to confirm the legitimacy of the analysis. Specifically, analyses revealed that all variables in the matrix were significantly correlated, yielding high percentage values ≥0.30. Additionally, most sampling adequacy coefficients exceeded 0.80, the determinant of the matrix was very low but not null, the anti-image coefficients were low in absolute values, the Kaiser-Meyer-Olkin (KMO) measurement was > 0.70, and the Bartlett's test of sphericity produced a significant result [22]. The number of components was determined using the Kaiser's criterion, which requires eigenvalues > 1 [23], and the Cattell scree-test on the sedimentation graph [24]. The percentage of variance explained for each item was calculated using h^2 ^values of communality. The items were distributed among the factors to which they connected most strongly, always with values w > 0.5 [24].

The internal consistency of each factor was calculated using the Cronbach's α, the item-rest discrimination coefficients and taking into account changes in α after eliminating each item. The Pearson correlation coefficient (r) was used to evaluate the discriminating power of dimensions in the BCSQ-12-SS and to examine the convergence between them and the MBI-SS dimension criterion. To further estimate the explanatory ability of the BCSQ-12-SS over the criterion, we calculated multiple correlation coefficients (R_y.123_) and multiple coefficients of determination (R^2^_y.123_).

Participants situated above the 75^th ^percentile (P_75_) for each dimension of the BCSQ-12-SS were considered to have "high scores", whereas those situated below the 75^th ^percentile were considered to have "low scores" [17,25,26]. Using simple binary logistic regression (LR) models to yield odds ratio (ORs) with a 95% confidence interval (CI), we conducted a bivariate analysis to assess the potential association between the burnout subtypes and socio-demographic and occupational variables of interest. The statistical significance of the association was assessed using the Wald test. The factors that showed significant values as a result of the bivariate analysis (p < 0.05) were included in a multivariate LR model to estimate the corresponding adjusted ORs and 95% CIs. The statistical significance of the adjusted ORs was assessed using the Wald test. The adjustment of each multivariate model was assessed using the Hosmer-Lemeshow χ^2 ^test, and according to the percentage of correctly classified cases, with a reference value of 0.5.

All contrasts were bilateral, with a significance level of p < 0.05. Data analysis was conducted using SPSS-15 and Epidat 3.1.

## Results

### Characteristics of the study participants

The sample of participants consisted of 314 students, who represented a RR of 83.07%. The participants were between 18 to 41 years of age (average = 22.05; SD = 3.57), with 70.70% of them being women. All participants described themselves as being white Europeans. Table 1 shows the socio-demographic and occupational characteristics for the entire sample and for each campus. Compared to students at Santiago, students at Huesca lived further away from the family home (p < 0.001), were more likely to live in shared flats (p = 0.002), were less likely to have received a scholarship (p = 0.011), and failed a higher percentage of subjects over the previous exam period (p = 0.040). Santiago and Huesca students were similar with regard to the rest of the socio-demographic and occupational variables.

**Table 1:** Socio-Demographic and Occupational Characteristics of Study Participants

	TOTAL (n = 314)	Huesca (n = 119)	Santiago (n = 195)	p
Age (years)				
< 20	109 (34.72)	42 (35.29)	67 (34.36)	0.989
20-22	116 (36.94)	44 (36.97)	72 (36.92)	
> 22	89 (28.34)	33 (27.74)	56 (28.72)	
Gender				
female	222 (70.70)	78 (65.55)	144 (73.85)	0.118
Stable Relationship				
no	158 (50.48)	59 (49.58)	99 (50.77)	0.952
Children				
without Children	300 (95.54)	112 (94.12)	188 (96.41)	0.340
Distance from family home (Km)				
< 75	110 (35.03)	24 (20.17)	86 (44.10)	**< 0.001**
75-150	103 (32.80)	23 (19.33)	80 (41.03)	
> 150	101 (32.17)	72 (60.50)	29 (14.87)	
Place of Residence				
with parents	38 (12.10)	7 (5.88)	31 (15.90)	**0.002**
dormitory	51 (16.24)	18 (15.13)	33 (16.92)	
shared flat	183 (58.28)	84 (70.59)	99 (50.77)	
private flat	42 (13.38)	10 (8.40)	32 (16.41)	
Receives a scholarship				
no	199 (63.38)	86 (72.27)	113 (57.95)	**0.011**
Family Support				
insufficient	20 (6.37)	5 (4.20)	15 (7.69)	0.202
good	74 (23.57)	24 (20.17)	50 (25.64)	
very good	220 (70.06)	90 (75.63)	130 (66.67)	
Weekly studying (hours)				
< 30	132 (42.04)	51 (42.86)	81 (41.54)	0.968
30-40	79 (25.16)	30 (25.21)	49 (25.13)	
> 40	103 (32.80)	38 (31.93)	65 (33.33)	
Failed subjects				
none	212 (67.92)	74 (62.18)	138 (70.77)	**0.040**
one	78 (24.57)	39 (32.77)	39 (20.00)	
two or more	24 (7.51)	6 (5.05)	18 (9.23)	
Job				
no	266 (84.71)	98 (82.35)	168 (86.15)	0.347
Year of study				
first	62 (19.75)	29 (24.36)	33 (16.92)	0.262
second	63 (20.06)	26 (21.85)	37 (18.97)	
third	60 (19.11)	21 (17.65)	39 (20.01)	
fourth	69 (21.97)	26 (21.85)	43 (22.05)	
fifth	60 (19.11)	17 (14.29)	43 (22.05)	

Frequencies, percentages (in parentheses), and p-values (χ^2 ^analysis) for the entire sample and grouped by campus

### Descriptive statistics

Table 2 shows the descriptive statistics for the items on the BCSQ-12-SS. Item n° 1 ("I think I invest more than is healthy in my commitment to my studies") showed the highest values (average = 4.07), whereas n° 9 ("I give up when faced with any difficulty in my tasks as a student") showed the lowest values (average = 1.85). The variability of the items presented SD values that ranged between 1.06 (for item n° 9) and 1.84 (for item n° 7: "I am endangering my health in pursuing good results in my studies"). Individual answers covered the entire range (from 1.00 to 7.00) of the scale, with the exception of item n° 9 (maximum value = 5.00).

**Table 2:** Factorial Weights, descriptive statistics, communalities, and coefficients of discrimination

	Components						
	1	2	3	Average	SD	h^2^	item-rest
1. I think I invest more than is healthy in my commitment to my studies	**0.76**	-0.04	-0.11	4.07	1.59	0.59	0.58
4. I neglect my personal life to pursue great accomplishments in studying	**0.81**	0.13	0.16	3.26	1.77	0.70	0.68
7. I am endangering my health in pursuing good results in my studies	**0.88**	0.12	0.07	2.98	1.84	0.79	0.78
10. I ignore my own needs to satisfy the requirements of my studies	**0.84**	0.09	0.16	2.98	1.75	0.73	0.73

3. When the results of my studies are not good at all, I stop making an effort	0.01	**0.77**	0.04	2.25	1.41	0.59	0.59
6. I give up in response to an obstacle in my studies	0.17	**0.78**	0.08	2.14	1.35	0.63	0.63
9. I give up when faced with any difficulty in my tasks as a student	-0.03	**0.81**	0.26	1.85	1.06	0.73	0.70
12. When the effort invested in studying is not enough, I give up	0.13	**0.80**	0.15	2.03	1.22	0.69	0.66

2. I would like to study something else that would be more challenging to my abilities	-0.06	-0.07	**0.80**	2.73	1.65	0.65	0.53
5. I feel that my current studies are hampering the development of my abilities	0.21	0.39	**0.71**	2.32	1.35	0.70	0.66
8. I would like to study something else in which I could better develop my talent	0.02	0.13	**0.86**	2.41	1.61	0.76	0.72
11. My studies do not provide me with opportunities to develop my abilities	0.22	0.32	**0.72**	2.37	1.45	0.68	0.64

Extraction Method: Principal Component Analysis with Varimax rotation. SD = standard deviation. Item-rest = coefficient of item-rest discrimination according to factorial solution. h^2 ^= communalities.

### Factorial validity

All of the items on the BCSQ-12-SS showed significant correlations among themselves (75.76% of the total of these correlations). Among the correlations, 42.42% were > 0.30. All sampling adequacy coefficients exceeded 0.75, with 66.67% being > 0.80. The determinant factor of the matrix showed a value of 0.004, and all anti-image coefficients showed absolute values close to 0. The Kaiser-Meyer-Olkin measurement was very good (KMO = 0.82) and Bartlett's test of sphericity produced a significant result (χ^2 ^= 1,695.11; gl. = 66; p < 0.001). Together, these considerations allowed us to legitimately conduct the EFA.

The EFA yielded a three-factor solution with no forcing necessary. The first component showed an eigenvalue of λ_1 _= 4.25 (explaining 35.43% of the variance), the second component showed an eigenvalue of λ_2 _= 2.34 (19.49%), and the third component showed an eigenvalue of λ_3 _= 1.64 (13.66%). The three components satisfied Kaiser criterion and the Cattell's scree-plot test, and explained 68.58% of the total variance. Table 2 shows the factorial weights and h^2 ^values. Items n° 1, 4, 7 and 10 loaded on the first component ("overload") with values ranging from 0.76 (item 1) to 0.88 (item 7). Items n° 3, 6, 9, and 12 loaded on the second component ("neglect"), with values ranging from 0.77 (item 3: "When the results of my studies are not good at all, I stop making an effort") to 0.81 (item 9). Items n° 2, 5, 8, and 11 loaded on the third component ("lack of development"), with values ranging from 0.71 (item 5: "I feel that my present studies are hampering the development of my abilities") to 0.86 (item 8: "I would like to study something else in which I could better develop my talent"). The h^2 ^values were high in all cases, with values ≥0.59.

### Reliability

Table 2 shows the item-rest coefficients that revealed the association between the items on the BCSQ-12-SS and their respective factor components. Values ranged from 0.53 (item 2: "I would like to study something else that would be more challenging to my abilities") to 0.78 (item 7: **"**I am endangering my health in pursuing good results in my studies"). Analysis of the internal consistency of the BCSQ-12-SS resulted in α-values that exceeded 0.80 for all dimensions (Table 3). In all cases, the elimination of each item one at a time decreased the value of alpha coefficients.

**Table 3:** Descriptive statistics, internal consistency, and correlations among the dimensions of the BCSQ-12-SS and MBI-SS

	Average	SD	1	2	3	4	5	6
BCSQ-12-SS								
1. Overload	3.32	1.45	(0.85)					
2. Lack of development	2.46	1.22	0.21*	(0.81)				
3. Neglect	2.07	1.01	0.20*	0.38*	(0.82)			
MBI-SS								
4. Exhaustion	2.70	1.50	0.59*	0.23*	0.25*	(0.90)		
5. Cynicism	1.39	1.18	0.27*	0.49*	0.36*	0.46*	(0.78)	
6. Efficiency	4.14	0.94	-0.02	-0.24*	-0.47*	-0.12*	-0.36*	(0.76)

* p < 0.001 (bilateral); Values α into brackets in the diagonal. SD = standard deviation

### Convergent-discriminant validity

Table 3 shows the results of the convergent-discriminant analysis of validity. The highest convergence values were found for the following pairs of dimensions: "overload"-"exhaustion" (r = 0.58, p < 0.001), "lack of development"-"cynicism" (r = 0.48, p < 0.001), and "neglect"-"efficiency" (r = 0.49; p < 0.001). Taken together, the BCSQ-12-SS dimensions explained 38.44% of the variation in "exhaustion" (R_y.123 _= 0.62; p < 0.001), 30.25% in "cynicism" (R_y.123 _= 0.55; p < 0.001), and 26.01% in "efficiency" (R_y.123 _= 0.51; p < 0.001). Discrimination was r = 0.21 (p < 0.001) for "overload" and "lack of development", r = 0.20 (p < 0.001) for "overload" and "neglect", and r = 0.38 (p < 0.001) for "lack of development" and "neglect".

### Socio-demographic and occupational risk factors

In Table 4, we present the results of the univariate analysis on the potential socio-demographic and occupational risk factors. Only the university campus was found to be significantly related to the status variable "lack of development". Specifically, Santiago's students, when compared with Huesca's students, showed an OR = 2.07 (95% CI = 1.16-3.70; p = 0.013). This variable managed to correctly predict 76.43% of cases. The variables "year of study" and "weekly hours spent on studying" produced significant results after the multivariate analysis on the status variable "overload". Specifically, fifth-year students, when compared with first-year students, showed an OR = 0.32 (95% CI = 0.11-0.95; p = 0.041), students who dedicated > 40 hours to their studies every week, when compared with those dedicating < 30 hours, showed an OR = 3.41 (95% CI = 1.63-7.11; p = 0.001), and students who dedicated 30-40 hours an OR = 2.93 (95% CI = 1.34-6.43; p = 0.007). The adjustment of the model was acceptable (χ^2 ^= 7.05; gl = 8; p = 0.531), with 78.29% correctly classified cases. Both "received a scholarship" and "failed subjects over the past four trimesters", presented significant results after the multivariate analysis on the status variable "neglect". Specifically, students who did not receive a scholarship, compared to those who received one, yielded an OR = 0.56 (95% CI = 0.32-0.99; p = 0.048), students who failed two or more subjects, when compared with those who passed everything, yielded an OR = 3.36 (95% CI = 1.32-8.57; p = 0.011), and students who failed a subject an OR = 2.11 (95% CI = 1.12-3.98; p = 0.021). The adjustment of the model was acceptable (χ^2 ^= 0.09; gl = 2; p = 0.956), with 78.01% correctly classified cases.

**Table 4:** Univariate analysis for 'overload' ("frenetic" subtype), 'lack of development' ("underchallenged" subtype), and 'neglect' ("worn-out" subtype)

	Overload				Lack of development				Neglect			
**Factor**	**high score** **(%)**	**low score** **(%)**	**raw OR** **(95% CI)**	***p***	**high score** **(%)**	**low score** **(%)**	**raw OR** **(95% CI)**	***p***	**high score** **(%)**	**low score** **(%)**	**raw OR** **(95% CI)**	***p***

Age (years)												
< 20	23 (21.11)	86 (78.90)	ref.		27 (24.77)	82 (75.23)	ref.		23 (21.10)	86 (78.90)	ref.	
20-22	28 (24.14)	88 (75.86)	1.19 (0.64-2.23)	0.587	28 (24.14)	88 (75.86)	0.97 (0.53-1.78)	0.912	17 (14.78)	98 (85.22)	0.65 (0.33-1.29)	0.219
> 22	20 (22.73)	68 (77.27)	1.10 (0.56-2.17)	0.784	18 (20.45)	70 (79.55)	0.78 (0.40-1.54)	0.474	27 (31.03)	60 (68.97)	1.68 (0.88-3.21)	0.115
Gender												
male	17 (18.48)	75 (81.52)	ref.		27 (29.35)	65 (70.65)	ref.		16 (17.58)	75 (82.42)	ref.	
female	54 (24.32)	168 (75.68)	1.42 (0.77-2.61)	0.260	47 (21.17)	175 (78.83)	0.65 (0.37-1.12)	0.122	52 (23.53)	169 (76.47)	1.44 (0.77-2.69)	0.249
Stable relationship												
yes	42 (27.10)	113 (72.90)	ref.		35 (22.58)	120 (77.42)	ref.		34 (21.94)	121 (78.06)	ref.	
no	28 (17.72)	130 (82.28)	0.58 (0.34-0.99)	**0.047**	38 (24.05)	120 (75.95)	1.09 (0.64-1.83)	0.759	33 (21.15)	123 (78.85)	0.96 (0.56-1.64)	0.867
Children												
none	65 (21.67)	235 (78.33)	ref.		72 (24.00)	228 (76.00)	ref.		65 (21.81)	233 (78.19)	ref.	
1 or more	6 (42.86)	8 (57.14)	2.71 (0.91-8.09)	0.064	2 (12.29)	12 (85.71)	0.53 (0.12-2.41)	0.410	3 (41.43)	11 (78.57)	0.98 (0.27-3.61)	0.973
Campus												
Huesca	33 (27.73)	86 (72.27)	ref.		19 (16.97)	100 (84.03)	ref.		22 (18.80)	95 (81.20)	ref.	
Santiago	38 (19.49)	157 (80.51)	0.63 (0.37-1.08)	0.090	55 (28.21)	140 (71.79)	2.07 (1.16-3.70)	**0.013**	46 (23.59)	149 (76.41)	1.33 (0.75-2.36)	0.322
Distance from family home (Km)												
< 75	20 (18.18)	90 (81.82)	ref.		30 (27.27)	80 (72.73)	ref.		26 (23.64)	84 (76.36)	ref.	
75-150	29 (28.16)	74 (71.84)	1.76 (0.92-3.37)	0.086	24 (23.30)	79 (76.70)	0.81 (0.44-1.51)	0.506	22 (21.57)	80 (78.43)	0.89 (0.47-1.69)	0.719
> 150	22 (22.22)	77 (77.78)	1.29 (0.65-2.53)	0.467	20 (20.20)	79 (79.80)	0.66 (0.35-1.29)	0.233	20 (20.41)	78 (79.59)	0.83 (0.43-1.60)	0.576
Dwelling												
with parents	5 (13.16)	33 (86.84)	ref.		13 (34.21)	25 (65.79)	ref.		11(29.73)	26 (70.27)	ref.	
college	14 (28.00)	36 (72.00)	2.57 (0.83-7.91)	0.101	8 (16.00)	42 (84.00)	0.37 (0.13-1.01)	0.051	8 (16.00)	42 (84.00)	0.45 (0.16-1.27)	0.130
Shared flat	36 (19.67)	147 (80.33)	1.62 (0.59-4.43)	0.351	41 (22.40)	142 (77.60)	0.56 (0.26-1.18)	0.127	40 (22.98)	142 (78.02)	0.67 (0.30-1.46)	0.311
Flat without sharing	16 (38.10)	26 (61.91)	4.06 (1.32-12.55)	0.015	11 (26.19)	31 (73.81)	0.68 (0.26-1.78)	0.435	9 (21.43)	33 (78.57)	0.65 (0.23-1.79)	0.399
Scholarship												
yes	26 (22.61)	89 (77.39)	ref.		34 (29.57)	81 (70.43)	ref.		32 (28.07)	82 (71.93)	ref.	
no	45 (22.61)	154 (77.39)	1.00 (0.58-1.73)	0.999	40 (20.10)	159 (79.90)	0.60 (0.35-1.02)	0.057	36 (18.18)	162 (81.82)	0.57 (0.33-0.98)	**0.043**
Family support												
insufficient	3 (15.00)	17 (85.00)	ref.		5 (25.00)	15 (75.00)	ref.		5 (25.00)	15 (75.00)	ref.	
good	24 (32.43)	50 (67.57)	2.72 (0.73-10.19)	0.137	21(28.38)	53 (71.62)	1.19 (0.38-3.68)	0.765	14 (18.92)	60 (81.08)	0.70 (0.22-2.25)	0.549
very good	44 (20.00)	176 (80.00)	1.42 (0.40-5.05)	0.591	48 (21.82)	172 (78.18)	0.84 (0.29-2.42)	0.743	59 (22.48)	169 (77.52)	0.87 (0.30-2.51)	0.797
Studying time (hours/week)												
< 30	15 (11.54)	115 (88.46)	ref.		29 (22.31)	101 (77.70)	ref.		33 (25.78)	96 (74.22)	ref.	
30-40	21 (27.63)	55 (72.37)	2.93 (1.40-6.11)	**0.004**	23 (30.26)	53 (69.74)	1.51 (0.80-2.87)	0.206	16 (21.05)	60 (78.95)	0.77 (0.39-1.51)	0.445
> 40	33 (32.67)	68 (67.33)	3.72 (1.86-7.34)	**< 0.001**	20 (19.80)	81 (80.20)	0.86 (0.45-1.63)	0.644	17 (16.83)	84 (83.17)	0.58 (0.30-1.12)	0.106
Failed Subjects												
no	49 (24.62)	50 (75.38)	ref.		40 (20.10)	159 (79.90)	ref.		34 (17.09)	165 (82.91)	ref.	
one	17 (23.61)	55 (76.39)	0.95 (0.50-1.78)	0.864	22 (30.56)	50 (69.44)	1.75 (0.95-3.22)	0.072	21 (30.00)	49 (70.00)	2.08 (1.11-3.91)	**0.023**
two or more	3 (13.64)	19 (86.36)	0.48 (0.14-1.70)	0.258	7 (31.82)	15 (68.18)	1.86 (0.71-4.85)	0.208	9 (40.91)	13 (59.09)	3.36 (1.33-8.49)	**0.010**
Work (hours/week)												
yes	13 (27.08)	35 (72.92)	ref.		10 (20.83)	38 (79.17)	ref.		14 (29.17)	34 (70.83)	ref.	
no	58 (21.89)	207 (78.11)	0.75 (0.38-1.52)	0.430	64 (24.15)	201 (75.85)	1.21 (0.57-2.57)	0.619	54 (20.53)	209 (79.47)	0.63 (0.32-1.25)	0.186
School Year												
first	16 (26.23)	45 (73.78)	ref.		16 (26.23)	45 (73.77)	ref.		17 (28.33)	43 (71.67)	ref.	
second	14 (22.22)	49 (77.78)	0.80 (0.35-1.83)	0.603	11 (17.46)	52 (82.54)	0.60 (0.25-1.41)	0.239	12 (19.05)	51 (81.95)	0.60 (0.26-1.38)	0.228
third	21 (35.00)	39 (65.00)	1.51 (0.70-3.30)	0.296	15 (25.00)	45 (75.00)	0.94 (0.41-2.12)	0.877	14 (23.33)	46 (76.67)	0.77 (0.34-1.75)	0.532
fourth	12 (17.39)	57 (82.61)	0.59 (0.25-1.38)	0.224	15 (21.74)	54 (78.26)	0.78 (0.35-1.75)	0.549	11 (16.18)	57 (83.82)	0.49 (0.21-1.15)	0.100
fifth	7 (11.67)	53 (88.33)	0.37 (0.14-0.98)	**0.046**	16 (26.67)	44 (73.33)	1.02 (0.46-2.29)	0.957	13 (21.67)	47 (78.33)	0.70 (0.30-1.61)	0.400

% refers to the percentage in each step. Raw OR: Odds Ratio resulting from bivariate analysis. CI: confidence interval. Ref. = reference category. 'High score' implies scores higher than the upper quartile of the scores observed in the sample', 'low score' implies scores lower than or equal to the upper quartile.

## Discussion

This is the first study that proposes an adaptation of the short version of the "Burnout Clinical Subtype Questionnaire" or BCSQ-12 [18] for possible application to students, by means of the "Burnout Clinical Subtype Questionnaire Student Survey" or BCSQ-12-SS. This adaptation showed good psychometric properties, with a factorial structure that replicated the original design. This provides evidence that favours the use of the questionnaire and opens the possibility for fast differention between students by means of clinical subtypes of burnout.

The main strength of the present study was its high level of participation, reflected by a high RR, which made it appropriate to perform our selected analytical procedures. Moreover, because the study was conducted on samples of dental students (with similar RRs) coming from two different institutions and two distinct autonomous communities, it becomes easier to generalise the results. Generalisation was also supported by the fact that respondents behaved similarly to non-respondents in terms of age, gender, and year of study. Finally, mistakes in transcription were corrected using an "external supervision process", that is, by an independent coder. At the same time, the fundamental shortcoming of the study had to do with its cross-sectional and correlational design. Such designs do not withstand the elaboration of contrasts that are etiological in nature, but only allow the identification of associated risk factors.

The participants were young, and the majority of them were female, did not have children, lived in shared flats, did not receive a scholarship, enjoyed good family support, and had not failed subjects over the previous exam period. The responses to the items on the BCSQ-12-SS covered a large range of the Likert scale and had good variability and a high correlation with one another; together, these characteristics allowed us to legitimately conduct an EFA [21,27]. The EFA yielded three components structure ("overload", "neglect", and "lack of development", in order of appearance) that explained a high percentage of the variance [22,23]. The reliability analysis showed very good results in all dimensions and for all items, which evidenced the precision of the instrument [28]. The convergence between the three components and the standard definitions of burnout was moderately high, especially for "overload"-"exhaustion", "lack of development"-"cynicism", and "neglect"-"inefficiency". An adequate discriminating validity was found when differentiating between the subtypes, which allows us to keep using the term burnout when identifying its various manifestations [28]. Thus, the BCSQ-12-SS represents an improvement on the standard definitions of the MBI for students by making a more specific characterisation of burnout possible. In addition to enabling the quick differentiation among clinical subtypes, the BCSQ-SS allows the evaluation and development of interventions tailored to the characteristics of each individual.

The variables "weekly hours spent on studying" and "year of study" were associated with "overload". "Overload" is a central property of the "frenetic" subtype, which is characterised by a great commitment and high ambitions to the point of overloading oneself to fulfil work requirements, or in this case, study requirements [14-16]. We observed that the more hours that students spend studying, the more likely it is for them to score high on "overload" and, hence, experience more severe levels of exhaustion. This result is in line with the definition of the profile and with the findings of past work on samples of workers [17,29-32]. Moreover, fifth-year students were less likely than first-year students to experience "overload". This is reasonable given that the fifth year is the last one of the university career. In other words, fifth-year students are those who are about to graduate, who would soon finish their studies, and who have had time to learn to manage the sources of stress affecting them.

The variable "campus" was associated with "lack of development". "Lack of development" is a central property of the "under-challenged" subtype, which is characterised by feelings of indifference, boredom, and the perceived lack of personal development to the point that one is considering other occupations that might better express one's talent [14-16]. In comparison to students at Huesca, students at Santiago were more likely to score high on "lack of development", and hence, on "cynicism". It has been proposed that certain characteristics of the task and types of occupation (and in particular, mechanical tasks [17]) could make it more likely for a person to develop burnout, specifically with regard to the "under-challenged" profile [33-35]. The differences found between groups could be due to the fact that the University of Santiago enrols many more students and has to give more importance to the most formal aspects of teaching.

The variables "failed subjects over the previous four trimesters" and "received a scholarship " were found to be associated with "neglect". "Neglect" is the central property of the "worn-out" subtype [14-16], which is characterised by feelings of losing control of study outcomes, a perceived lack of recognition of one's efforts and the tendency to give up responsibilities. In particular, "neglect" results when one adopts passive, inefficient strategies to cope with obstacles; doing so leads to a reduced perceived level of efficacy and the tendency for a person to "throw in the towel" when encountering difficulties [36-40]. It has also been proposed that organisational rigidity of institutions, including universities, could influence the process by taking away a person's commitment to tasks [17]. In this context, it is easy to understand that low return on investment in the first part of the course year could result in subsequent "neglect". Finally, students who had received a scholarship, compared to those who had not received one, were more likely to score high in "neglect". This result may seem contradictory; however this is not the case if we consider that in Spain academic performace (together with family income) determines the qualification of scholarships for the following academic year, not the present one. In other words, students on scholarships receive a grant without having to justify it in the same year. From the exchange perspective, students on scholarships appreciated that there would be more gain in choosing a passive coping strategy in the face of difficulties in their studies. The way in which having received the scholarship is associated with more neglectful behaviour is complex, and it may be affected by both socio-cultural and educational factors owing to families' economic differences and education owing to the conditions under which the scholarship awarding system operates.

## Conclusions

The findings of this study are interesting because they reinforce and compliment the results obtained in previous studies that had studied burnout from a different occupational perspective. The BCSQ-12-SS represents an improvement on our understanding of burnout by enabling us to classify students affected by burnout into clinical subtypes, and by making it easier to understand the particular idiosyncrasies of individuals suffering from burnout. The associations observed between socio-demographic variables and the different subtypes enable quicker identification. Such associations can also be used to establish hypotheses that are etiological in nature, because they abide by the premise of temporal precedence in the action sequence [41]. Taken together, the findings of this study pave the way to the development of interventions that are tailored to the specific characteristics of each case of burnout according to the type of malaise experienced. Specific interventions are in particular demand for populations that are highly affected by burnout syndrome, such as dentists. Moreover, specific interventions will improve the efficiency of the few treatments that are currently available [42,43]. In fact, considering the economic and health implications that can be derived from this new understanding [44], the findings of this study could even be applied at the prevention level, in the education of students itself.

## Competing interests

The authors declare that they have no competing interests.

## Authors' contributions

JM-M, FM, MG and JG-C conceived the project. AR, MC, and IT collected the data. JM-M and MG conducted the statistical analysis. All authors interpreted the results, drafted the manuscript and read and approved the final manuscript.

## Pre-publication history

The pre-publication history for this paper can be accessed here:

http://www.biomedcentral.com/1472-6920/11/103/prepub

## Supplementary Material

Additional file 1 **"Cuestionario de Subtipos Clínicos de Burnout, Versión Estudiantes" (BCSQ-12-SS)**. This file contains the Spanish version of the BCSQ-12-SS. Click here for file

Additional file 2 **"Burnout Clinical Subtype Questionnaire, Student Survey" (BCSQ-12-SS)**. This file contains the English version of the BCSQ-12-SS. Click here for file
