# Supplementary material for: Burnout syndrome among dental students: a short version of the "Burnout Clinical Subtype Questionnaire" adapted for students (BCSQ-12-SS)
Source: BMC Med Educ. 2011 Dec 12;11:103. doi: 10.1186/1472-6920-11-103 (PMC3273439; doi:10.1186/1472-6920-11-103)
Supplement: Additional file 1 — "Cuestionario de Subtipos Clínicos de Burnout, Versión Estudiantes" (BCSQ-12-SS). This file contains the Spanish version of the BCSQ-12-SS. [file 1472-6920-11-103-S1.DOC]

**ADDITIONAL FILES**

**Additional File 1**

“Cuestionario de Subtipos Clínicos de Burnout, Versión Estudiantes” (BCSQ-12-SS)

A continuación se presentan una serie de enunciados que indican vivencias que puede experimentar en su actividad como estudiante. Lea cada frase con atención y señale con una X la opción que mejor represente cómo se siente, lo que hace o lo que piensa respecto a su actividad como estudiante. No existen respuestas correctas o incorrectas. Por favor, **NO DEJE NINGUNA RESPUESTA SIN CONTESTAR.**

**1** Totalmente en desacuerdo

**2** Muy en desacuerdo

**3** En desacuerdo

**4** Indeciso

**5** De acuerdo

**6** Muy de acuerdo

**7** Totalmente de acuerdo

|  | **1 2 3 4 5 6 7** |
| --- | --- |
| 1. Creo que invierto más de lo saludable en mi dedicación al estudio | O O O O O O O |
| 2. Me gustaría estudiar alguna otra cosa que planteara mayores desafíos a mi capacidad | O O O O O O O |
| 3. Cuando los resultados de mis estudios no salen del todo bien dejo de esforzarme | O O O O O O O |
| 4. Descuido mi vida personal al perseguir grandes objetivos en el estudio | O O O O O O O |
| 5. Siento que mis actuales estudios son un freno para el desarrollo de mis capacidades | O O O O O O O |
| 6. Me rindo como respuesta a las dificultades en el estudio | O O O O O O O |
| 7. Arriesgo mi salud en la persecución de buenos resultados en el estudio | O O O O O O O |
| 8. Me gustaría estudiar otra cosa en la que pudiera desarrollar mejor mi talento | O O O O O O O |
| 9. Abandono ante cualquier dificultad en las tareas de mis estudios | O O O O O O O |
| 10. Ignoro mis propias necesidades por cumplir con las demandas del estudio | O O O O O O O |
| 11. Mis estudios no me ofrecen oportunidades para el desarrollo de mis aptitudes | O O O O O O O |
| 12. Cuando el esfuerzo invertido en el estudio no es suficiente, me doy por vencido | O O O O O O O |
