# Supplementary material for: Burnout syndrome among dental students: a short version of the "Burnout Clinical Subtype Questionnaire" adapted for students (BCSQ-12-SS)
Source: BMC Med Educ. 2011 Dec 12;11:103. doi: 10.1186/1472-6920-11-103 (PMC3273439; doi:10.1186/1472-6920-11-103)
Supplement: Additional file 2 — "Burnout Clinical Subtype Questionnaire, Student Survey" (BCSQ-12-SS). This file contains the English version of the BCSQ-12-SS. [file 1472-6920-11-103-S2.DOC]

**Additional File 2**

**Tittle**: “Burnout Clinical Subtype Questionnaire, Student Survey” (BCSQ-12-SS)

Below are some statements that relate to experiences in student life. Read each sentence carefully and mark with an X the option that best represents how you feel or what you think regarding your experiences as a student. There are neither right nor wrong answers. Please, **DO NOT LEAVE ANY QUESTIONS UNANSWERED.**

**1** Totally disagree

**2** Strongly disagree

**3** Disagree

**4** Undecided

**5** Agree

**6** Strongly agree

**7** Totally agree

|  | **1 2 3 4 5 6 7** |
| --- | --- |
| 1. I think I invest more than is healthy in my commitment to my studies | O O O O O O O |
| 2. I would like to study something else that would be more challenging to my abilities | O O O O O O O |
| 3. When the results of my studies are not good at all, I stop making an effort | O O O O O O O |
| 4. I neglect my personal life due to pursuing great objectives in studying | O O O O O O O |
| 5. I feel that my current studies are hampering the development of my abilities | O O O O O O O |
| 6. I give up in response to an obstacle in my studies | O O O O O O O |
| 7. I am endangering my health in pursuing good results in my studies | O O O O O O O |
| 8. I would like to study something else in which I could better develop my talent | O O O O O O O |
| 9. I give up when faced with any difficulty in my tasks as a student | O O O O O O O |
| 10. I ignore my own needs to satisfy the requirements of my studies | O O O O O O O |
| 11. My studies do not provide me with opportunities to develop my abilities | O O O O O O O |
| 12. When the effort invested in studying is not enough, I give up | O O O O O O O |
